# Impact of Kiwifruit Consumption on Cholesterol Metabolism in Rat Liver: A Gene Expression Analysis in Induced Hypercholesterolemia

**DOI:** 10.3390/nu16233999

**Published:** 2024-11-22

**Authors:** Abdolvahab Ebrahimpour Gorji, Anna Ciecierska, Hanna Leontowicz, Zahra Roudbari, Tomasz Sadkowski

**Affiliations:** 1 Department of Physiological Sciences, Institute of Veterinary Medicine, Warsaw University of Life Sciences, 02-776 Warsaw, Poland; abdolvahab_ebrahimpourgorji1@sggw.edu.pl (A.E.G.); anna_ciecierska@sggw.edu.pl (A.C.); hanna_leontowicz@sggw.edu.pl (H.L.); 2 Department of Animal Science, Faculty of Agriculture, University of Jiroft, Jiroft 78671-55311, Iran; roudbari.zahra@ujiroft.ac.ir

**Keywords:** hypercholesterolemia, *Actinidia arguta*, kiwifruit, liver steatosis, lipid metabolism

## Abstract

Background/Objectives: Cholesterol is vital in various bodily functions, such as maintaining cell membranes, producing hormones, etc. However, imbalances, like hypercholesterolemia, can lead to diseases such as cancer, kidney disease, non-alcoholic fatty liver disease, and cardiovascular conditions. This study explores the impact of kiwifruit consumption, specifically *Actinidia arguta* cultivar Geneva and *Actinidia deliciosa* cultivar Hayward, on cholesterol and lipid metabolism in rat liver. Methods: Rats were divided into groups: a 1% cholesterol control group (Ch), a 5% Geneva kiwifruit-supplemented group (ChGENE), and a 5% Hayward kiwifruit-supplemented group (ChHAYW). Gene expression was analyzed using Gene Spring v.14. Gene ontology, pathway analysis, miRNA, and transcription factor prediction were performed using DAVID, Reactome, and miRNet. In addition, we used Agilent Literature Search software to gain further insights. Results: Statistical analysis identified 72 genes in ChGENE-Ch and 2 genes in ChHAYW-Ch comparison. Key genes involved in cholesterol metabolism pathways, including *PCSK9*, *SCD1*, *SLC27A5*, *HMGCR*, and *DHCR24*, showed lower expression in the kiwifruit-supplemented groups. The genes mentioned above showed lower expression in the kiwifruit-supplemented group, probably contributing to the liver lipid level reduction. Further analysis identified miRNA-26a, miRNA-29a/b/c, miRNA-33a/b, and miRNA-155 targeting hub genes. Conclusions: Our findings suggest that dietary supplementation with kiwifruit, particularly the Geneva cultivar, reduces fat accumulation in the liver of rats with hypercholesterolemia, likely through downregulation of critical genes involved in cholesterol metabolism. These studies highlight the potential of kiwifruit as a part of a dietary strategy to manage cholesterol levels.

## 1. Introduction

Nonalcoholic fatty liver disease (NAFLD) ranges from simple hepatic steatosis to nonalcoholic steatohepatitis (NASH), involving lipid accumulation, liver cell damage, and inflammation. Linked to metabolic disorders like obesity and diabetes, the progression mechanism to NASH is unclear. No FDA-approved treatment exists, and lifestyle changes remain the primary intervention [1,2].

The lipid constituents within biological membranes play a crucial role in maintaining the standard functionality of cells. Any irregularities in their distribution or metabolism significantly affect individual cells and whole organisms. Lipid metabolism abnormalities, including hypercholesterolemia, might lead to cancer [3] and kidney, non-alcoholic fatty liver [4], cardiovascular [5], and other diseases. Other functions, including phosphor-inositide-mediated signaling, have similarly undergone several studies. Discoveries in the field have unraveled new insights into the regulation mechanisms of these pathways, which shed light on their spatial and temporal regulation in previously unknown ways [6]. Cholesterol is a fundamental constituent of natural fat substances. It plays an essential role in the body because it is vital for cell membrane function, nerve fiber insulation, kidney functions, sex hormones, bile acid production, etc. However, imbalances such as hypercholesterolemia lead to the incidence of hypertension due to occlusions of the peripheral vessels [7]. The sources contributing to cholesterol levels include carob fibers, *n*-3 fatty acids, and cholesterol-lowering phytosterols [8]. However, the relation of cholesterol with tumor development could be more complex since variable results have been noted about the association of hypercholesterolemia with cancer [9].

Consuming kiwifruit offers a range of health benefits. Regular intake of golden kiwifruit has been associated with reduced body fat mass, better blood pressure regulation, and managing inflammatory responses in overweight and obese individuals [10]. Kiwifruit is a commendable provider of antioxidants, exhibiting a favorable standing in terms of its overall antioxidant capability compared to other frequently ingested fruits such as grapefruit, apples, and pears [11]. *Actinidia deliciosa*, the Hayward cultivar, is the most popular variety of kiwi. In contrast, the awareness surrounding other species like *Actinidia eriantha* (cultivar Bidan) and *Actinidia arguta* (commonly referred to as mini kiwi or hardy kiwifruit) is comparatively limited [12]. An investigation into the impact of kiwifruit on cholesterol levels in rats found that when rats were fed a diet containing 1% cholesterol and 16% fat for six weeks, there was a notable increase in total cholesterol and triglyceride levels in their serum and liver. However, groups that received kiwifruit and avocado (fruit and seed) experienced significant reductions in serum total cholesterol, triglycerides, and low-density lipoprotein (LDL)-C levels. Moreover, these groups exhibited lowered levels of total cholesterol and triglycerides in their liver. This study suggests that incorporating kiwifruit and avocado into the diet may provide protection and have beneficial effects in reducing the risks of atherosclerosis and cardiovascular disease in hypercholesterolemic rats [13]. In addition, the study indicates that fermented kiwifruit juice has a cholesterol-regulating impact on mice suffering from hyperlipidemia [14]. Another study examined the effect of kiwifruit seed oil (KSO) on hepatic fat metabolism in obese mice. The results revealed that administering KSO decreased the expression of genes associated with lipid synthesis, such as peroxisome proliferator-activated receptor gamma (*PPARG*) and fatty acid synthase (*FAS*). Simultaneously, it upregulated the expression of genes related to fatty acid β-oxidation, including peroxisome proliferator-activated receptor alpha (*PPARA*), carnitine palmitoyltransferase 1A (*CPT1A*), and *CPT1B*, along with the thermogenesis-related gene uncoupling protein 2 (*UCP2*) in the liver. Furthermore, this study highlighted that mice on a high-fat diet exhibited significant suppression in both protein and mRNA levels of *PPARA*, a crucial lipid metabolism gene. However, after 12 weeks of KSO administration, the expression of *PPARA* was gradually restored to normal levels in a dose-dependent manner [15].

Previously, a study focused on evaluating the potential health advantages of Polish hardy kiwifruit, also known as mini kiwifruit (*Actinidia arguta*) Bingo, M1, Anna, Weiki, Jumbo, and Geneva, compared to two Asian varieties (Hayward and Bidan) in rats with induced hypercholesterolemia. The results revealed that including hardy kiwifruit in the diet enhanced the lipid profile, increased antioxidant capacity, and reduced liver damage and aortic lesions in rats consuming a cholesterol-rich diet [16]. This study investigated the relationship between the consumption of kiwifruit Geneva and Hayward varieties and cholesterol and lipid metabolism. The main objective of the present study, as a continuation of previous research, was to explore the potential molecular mechanisms linked with the effect of Geneva and Hayward kiwifruit supplementation on gene/miRNA expression profiles, most notably in the prevention of hepatic steatosis in rats exposed to an atherogenic diet.

## 2. Materials and Methods

### 2.1. Study Design

Leontowicz et al. (2016) reported that histological and biochemical changes were observed in the livers and aortic arch and the blood plasma of rats fed an atherogenic diet supplemented with freeze-dried kiwifruit, especially the Geneva cultivar. Their study compared the level of hepatocyte fat content with normal liver tissue. Based on the Leontowicz et al. study, three groups were chosen for microarray analysis of gene expression in hepatic tissue. The Leontowicz results demonstrated that the group subjected to a high-cholesterol diet (1%) exhibited significantly elevated lipid content in rat liver cells, primarily due to excessive dietary cholesterol intake. In contrast, the liver section from the Hayward cultivar-consuming group showed a notable reduction in fat accumulation within hepatocytes; however, liver steatosis persisted at an advanced stage. Furthermore, the liver tissue from the Geneva cultivar-consuming group closely resembles that of the negative control group (normal liver without cholesterol loading), where only a few hepatocytes exhibited fat deposits [17]. This finding underscores the protective effect of bioactive substances from kiwifruit varieties studied, particularly *Actinidia arguta*, cultivar Geneva, on liver function when exposed to a high-cholesterol diet.

Building on these findings and liver samples from the same rats, this study further analyzed gene expression in the liver tissue of rats exposed to three dietary regimens. The II Local Ethics Commission of Warsaw University of Life Sciences—SGGW, Poland, accepted a study involving male Wistar rats (Leontowicz et al., 2016) [17]. The rats were divided into three groups: the Ch group, which received a standard semi-synthetic diet containing 1% cholesterol (the control group); the ChGENE group, which was fed the control diet supplemented with 5% lyophilized kiwifruit cultivar Geneva; and the ChHAYW group, which was given the control diet supplemented with 5% lyophilized kiwifruit cultivar Hayward. Each dietary condition was implemented with eight biological replicates, ensuring a robust and dependable dataset for subsequent analysis and comparisons (*n* = 24).

### 2.2. Sample Collection and RNA Extraction

**Figure 1:**
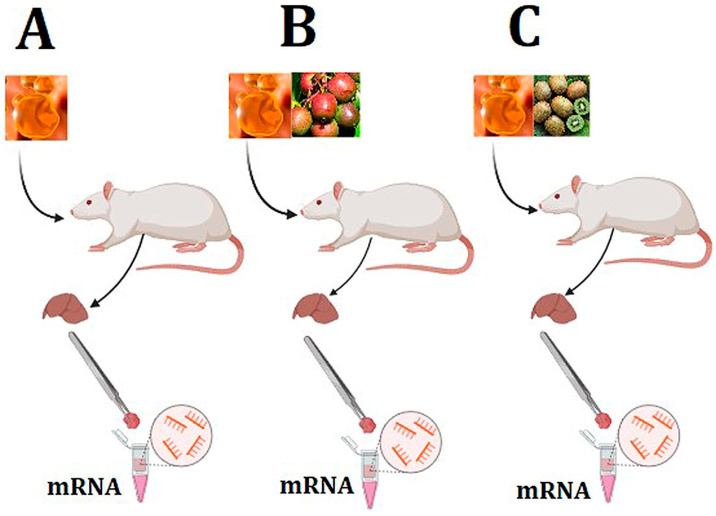
Schematic showing the main steps of the experimental setup. (**A**)—Ch control group fed with a semi-synthetic diet with the addition of 1% cholesterol, (**B**)—ChGENE group fed a semi-synthetic diet with the addition of 1% cholesterol and 5% freeze-dried fruits of *A. arguta* cultivar Geneva, (**C**)—ChHAYW group fed with diet semi-synthetic with the addition of 1% cholesterol and 5% freeze-dried *A. deliciosa* cultivar Hayward.

After a 42-day experimental period, the rats were dissected, and liver samples were promptly collected and stored at −80 °C until analysis. To extract total RNA, the protocol provided with the miRNANeasy Mini Kit was followed (Qiagen, Germantown, MD, USA). Subsequently, the quantity of RNA was measured using a NanoDrop (NanoDrop Technologies, Wilmington, DE, USA), and RNA quality and integrity were checked using a BioAnalyzer 2100 (Agilent Technologies, Santa Clara, CA, USA). In our quest to maintain the highest data quality standards, only RNA samples with a RIN (RNA Integrity Number) value equal to or exceeding 9.2 have been selected for analysis (Figure 1).

### 2.3. Microarray Analysis

The gene expression profile was analyzed using an Agilent-028279 SurePrint G3 Rat GE 8x60K Microarray GPL14797 (Agilent Technologies, USA) and an Agilent One-Color Microarray-Based Gene Expression Analysis kit (Agilent Technologies, USA). Probe labeling, hybridization, signal detection, and data extraction were conducted as described in a previous paper [18]. The data obtained from the microarray experiment were deposited in the National Center for Biotechnology Information Gene Expression Omnibus database (NCBI GEO) with accession number GSE79715. The statistical analysis was performed using Gene Spring v.14 software (Agilent Technologies, USA). A Student’s *t*-test with a significance threshold set at *p* < 0.05 and FDR < 0.05 was applied to assess the statistical significance of differences. A fold change (FC) greater than 1.3 was a criterion for relevance.

### 2.4. Real-Time qPCR Analysis

The real-time qPCR technique has been used to verify the transcriptomic analysis results. Five DEGs were selected to validate microarray results: proprotein convertase subtilisin/kexin 9 (*PCSK9*), 24-dehydrocholesterol reductase (*DHCR24*), 3-hydroxy-3-methylglutaryl-coenzyme A reductase (*HMGCR*), proline dehydrogenase 1 (*PRODH*), and stearoyl-CoA desaturase-1 (*SCD1*). Glyceraldehyde 3-phosphate dehydrogenase (*GAPDH*) was used as a reference gene. Primers were designed and validated, and cDNA were synthesized and the analysis was conducted according to Szcześniak et al.’s (2016) protocol [19] (Table 1). Assays were performed in triplicate using a Stratagene Mx3005p thermal cycler (Stratagene, San Diego, CA, USA) according to the following protocol: (1) pre-incubation for 2 min at 95 °C; (2) amplification (40 cycles) containing denaturation at 95 °C for 5 s and annealing at a temperature specified in Table 1 for 15 s; (3) dissociation curve with setting as follows: denaturation at 95 °C for 0 s, annealing at a temperature specified in Table 1, continuous melting up to 95 °C for 0 s (slope = 0.1 °C/s), and cooling at 40 °C for 30 s. The level of mRNA expression was calculated using GenEx 6.0 (MultiD Analyses, Västra Frölunda, Sweden) and is presented as ΔΔCt [20]. GraphPad Prism 5 (GraphPad Software Inc., Boston, MA, USA) software was used for data visualization. The results have been presented as mean ± standard error (SEM) and were marked as statistically significant with * for *p*  <  0.05, ** for *p*  <  0.01, and *** for *p*  <  0.001. RT-qPCR analysis was conducted using a standardized approach [21].

### 2.5. Pathway, Gene Ontology, and Literature Analysis

We analyzed differentially expressed genes and delved into their gene ontology (GO). To conduct the GO analysis, we utilized the Database for Annotation, Visualization, and Integrated Discovery (DAVID), accessed at http://david.ncifcrf.gov/ (accessed on 20 July 2024). In addition, we considered Reactome (https://reactome.org/) (accessed on 20 July 2024) for pathway analysis in our investigation.

To define the links between target genes and proteins and to visualize these relations, we employed the Agilent Literature Search software version 3.1.1 as an extension for Cytoscape. This tool uses multiple text-based search engines to obtain information. It is helpful for biomedical text mining and biological network analysis, as it turns the identified molecules or topics into networks by converting the relationship. Throughout our analysis, we rigorously filtered out any false-positive interaction data from the retrieval results. Subsequently, we imported the gene/protein interaction relationships into Cytoscape 3.9.1 for visualization and further exploration.

### 2.6. MiRNA and Transcription Factor Prediction

The online software miRNet, available at (https://www.mirnet.ca/) (accessed on 26 July 2024), has been used to identify potential miRNAs significant for cholesterol metabolism genes. MiRNet is an online tool and database that explores miRNA-target interactions across species. We also predict transcription factors that may regulate the expression of these genes. A human database was applied instead of a rat database to extend our research on liver-expressed miRNAs.

## 3. Results

### 3.1. Identification of Differentially Expressed Genes

**Figure 2:**
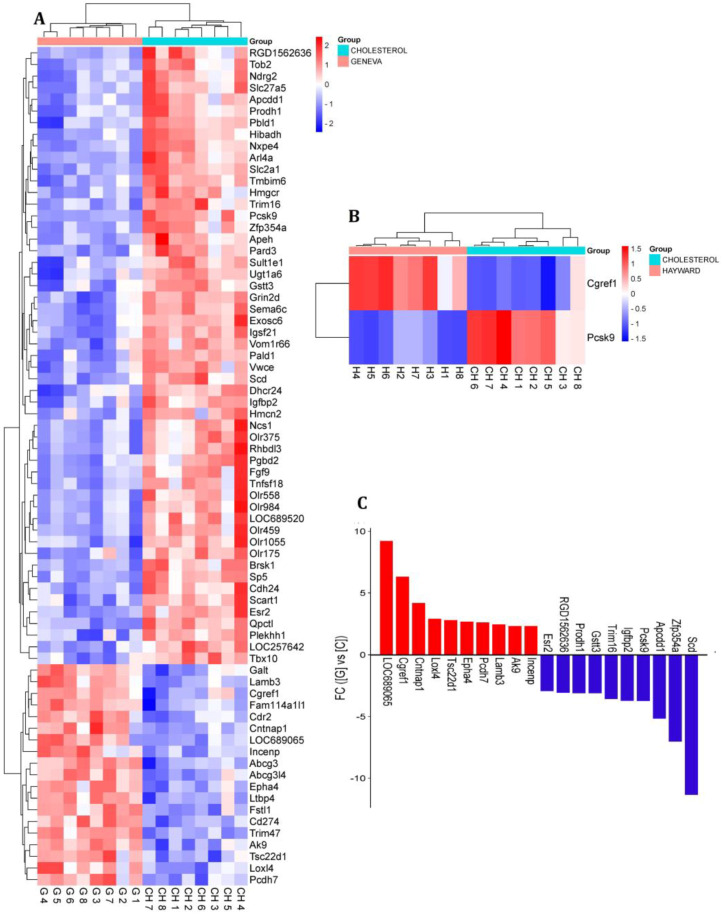
The gene expression profiles of ChGENE and ChHAYW in comparison to Ch. (**A**) Heatmap depicting the gene expression of ChGENE. (**B**) Heatmap illustrating the gene expression of ChHAYW. (**C**) Presentation of the top ten upregulated and downregulated genes in ChGENE compared to Ch.

**Figure 3:**
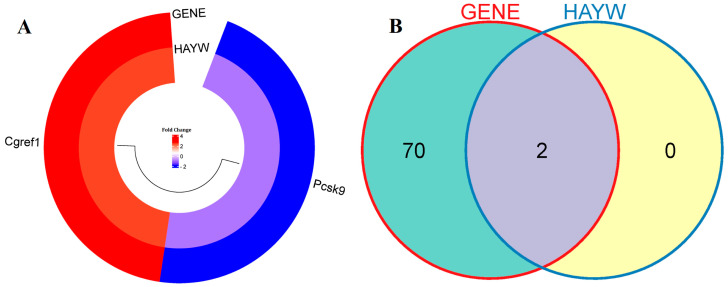
(**A**) The circular heatmap visually conveys the proportion of shared gene expression between ChGENE and ChHAYW. (**B**) The Venn diagram illustrates the gene expression count in ChGENE and ChHAYW.

This study compared two gene expression datasets to identify differentially expressed genes (DEGs) between the ChGENE/ChHAYW and Ch control group. In the ChGENE-Ch comparison, we observed that 72 genes displayed significant differential expression (fold change ≥ 1.3, *p*-value < 0.05), with 19 genes being upregulated and 53 genes being downregulated. Within the ChHAYW-Ch comparison, we identified two significant genes, one downregulated and one upregulated. Ten top upregulated genes in the ChGENE-Ch group, including *LOC689065*, *CGREF1*, *CNTNAP1*, *LOXL4*, *TSC22D1*, *EPHA4*, *PCDH7*, *LAMB3*, *AK9* and *INCENP*, and ten top downregulated genes, including *ESR2*, *RGD1562636*, *PRODH1*, *GSTT3*, *TRIM16*, *IGFBP2*, *PCSK9*, *APCDD1*, *ZFP354A*, and *SCD1*, are shown in Figure 2 and in Supplementary File S1. Notably, there was an overlap of two genes that were expressed in both comparisons (Figure 3).

### 3.2. Real-Time qPCR Validation

**Figure 4:**
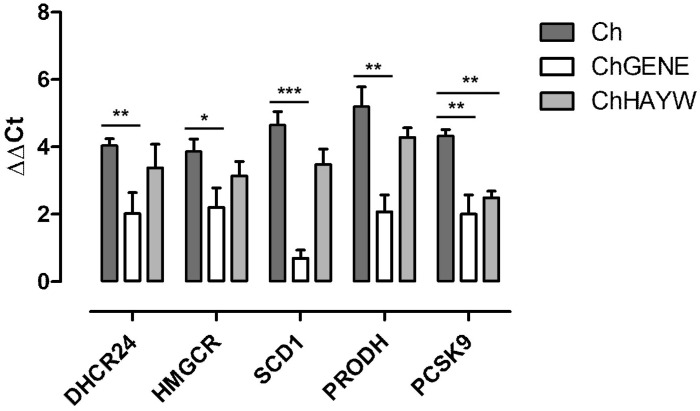
Quantitative expression analysis of genes related to lipids and cholesterol metabolism by Real-time qPCR. Statistical significance is shown as * *p* < 0.05, ** *p* < 0.01, and *** *p* < 0.001.

Five genes implicated in lipid and cholesterol metabolism processes were chosen for validation: *PCSK9*, *DHCR24*, *HMGCR*, *PRODH*, and *SCD1*. Statistically significant changes in the expression of all examined DEGs were detected using the Real-time qPCR technique, which corresponded with the results obtained from microarray analyses and were downregulated in ChGENE compared to Ch. *PCSK9* was also downregulated in ChHAYW compared to Ch (Figure 4).

### 3.3. GO and Pathway Analysis

**Figure 5:**
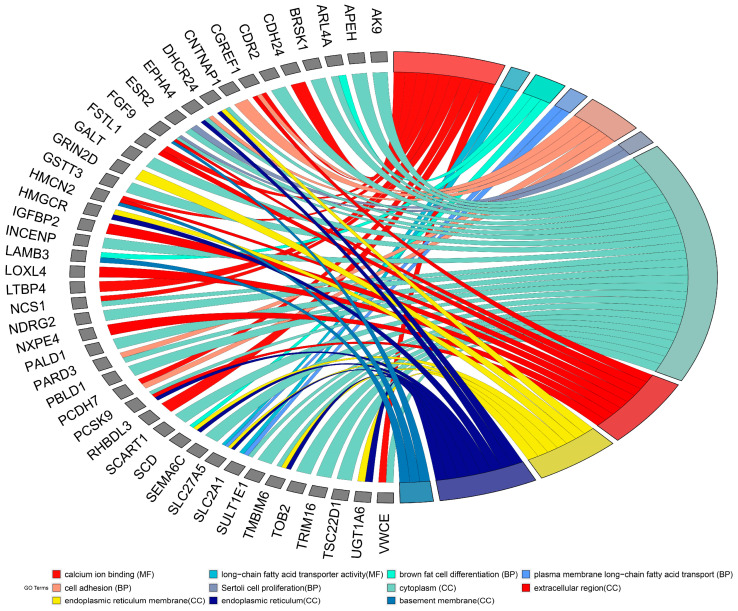
The chord plot represents the relationship between GO terms and genes exhibiting significant expression changes under the ChGENE and ChHAYW treatments.

DEGs were uploaded to DAVID to identify GO and pathways. GO analysis for the ChGENE-Ch comparison showed that DEGs were significantly enriched in biological processes (BP), including brown fat cell differentiation, plasma membrane long-chain fatty acid transport, and cell adhesion. For molecular function (MF), the DEGs were enriched in calcium ion binding and long-chain fatty acid transporter activity. Concurrently, the cell component (CC) analysis has also shown that the DEGs were significantly enriched in the cytoplasm, extracellular region, and endoplasmic reticulum membrane (Figure 5).

**Figure 6:**
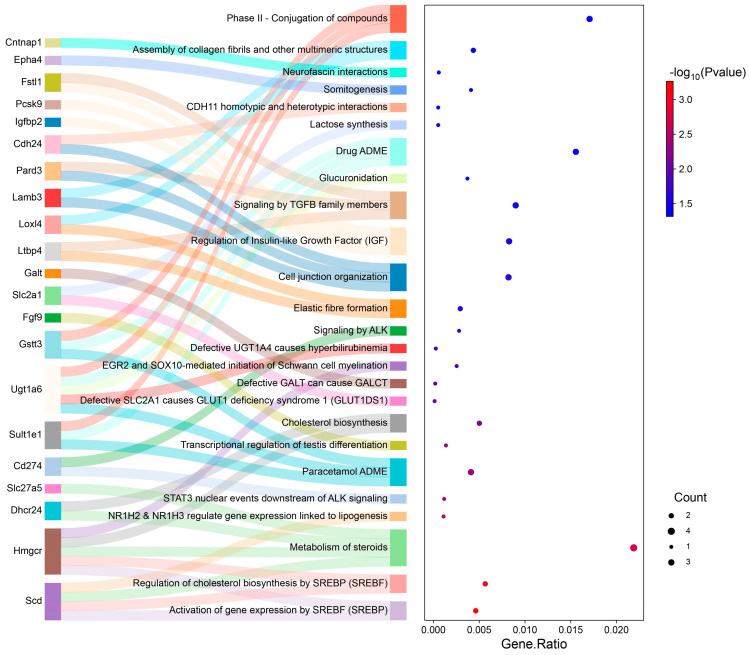
A Sankey plot depicting genes within individual pathways (**left**) and a dot plot where the size of the dots corresponds to the number of genes; the dot’s color signifies the *p*-values (**right**) for the ChGENE treatment.

In the context of our study, a subsequent analysis involving Reactome pathway enrichment was conducted for the ChGENE-Ch comparison. The analysis highlighted the top four enriched pathways: Activation of gene expression by SREBF; Regulation of cholesterol biosynthesis by SREBF; Metabolism of steroids; and NR1H2 and NR1H3 regulating gene expression linked to lipogenesis. This enrichment was primarily driven by specific enzymes and transport proteins engaged in lipid metabolism, including *SCD1*, *DHCR24*, *HMGCR*, and *SLC27A5* (Figure 6).

**Figure 7:**
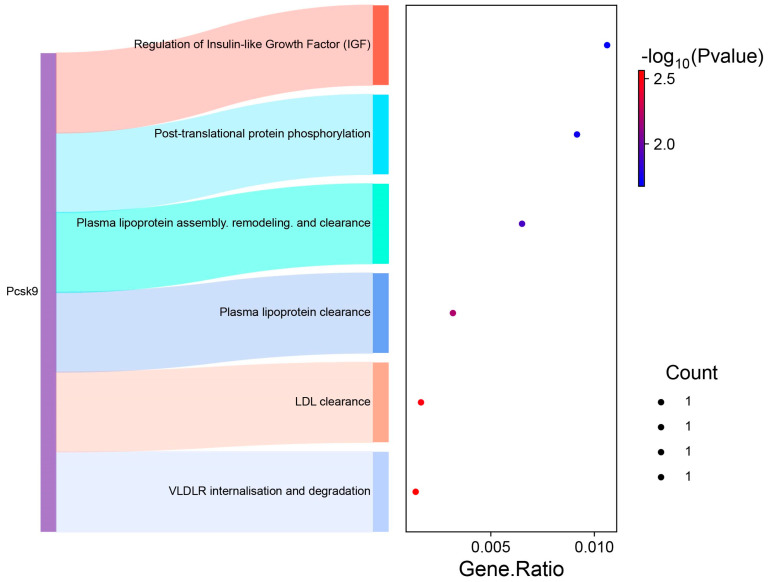
A Sankey plot depicting genes within individual pathways (**left**) and a dot plot where the size of the dots corresponds to the number of genes and the color of the dots signify the *p*-values (**right**) for the ChHAYW treatment.

For the ChHAYW-Ch comparison, the pathway analysis highlighted the top four pathways: VLDLR internalization and degradation; LDL clearance; Plasma lipoprotein clearance; and Plasma lipoprotein assembly, remodeling, and clearance, enriched by a gene common between ChGENE and ChHAYW, namely *PCSK9* (Figure 7).

### 3.4. Identification of Hub Genes Based on Literature Analysis

**Figure 8:**
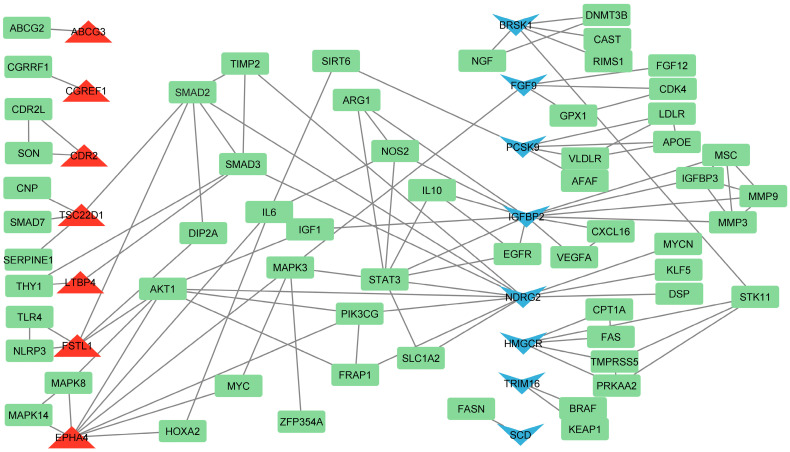
Agilent Literature Search network analysis between DEGs and other critical genes governing cholesterol and lipid metabolism. Green squares denote genes identified by Agilent Literature Search as engaged in lipid metabolism; red triangles signify upregulated DEGs; blue V-shaped symbols signify downregulated DEGs.

Using the Agilent Literature Search software (version 3.1.1) as a Cytoscape plugin, we have uncovered connections and interactions within the network. Our findings reveal that *PCSK9* is downregulated in ChGENE-Ch/ChHAYW-Ch comparisons and, according to Agilent Literature Search network analysis, is connected to *APOE*, *LDLR*, *VLDLR*, *AFAF*, and *SIRT6*. Furthermore, we have observed interactions among two DEGs from our study, downregulated *FGF9* and upregulated *EPHA4*, with the *MAPK3* gene, which plays a role in lipid metabolism. Upregulated *FSTL1* and the previously mentioned *EPHA4* may interact with *AKT1*, suggesting regulatory relationships or functional links between these genes in cholesterol metabolism (Figure 8).

### 3.5. Prediction of miRNA and Transcription Factors

**Figure 9:**
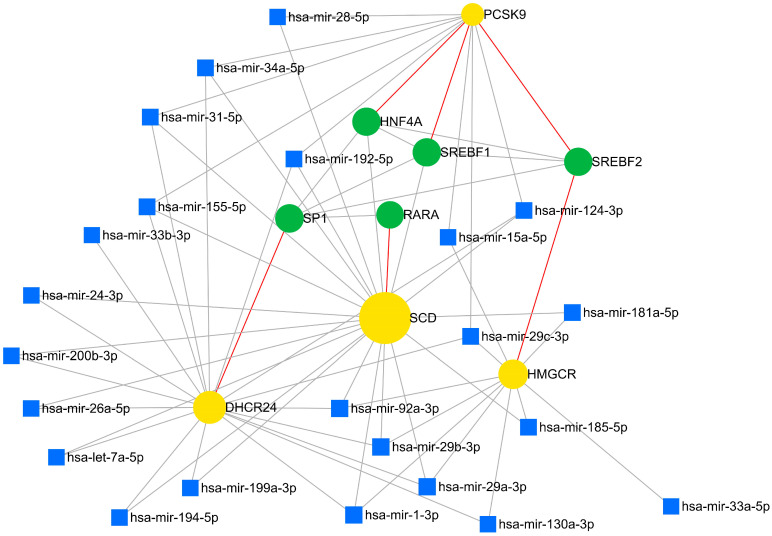
The interaction network of predicted miRNAs and their corresponding target DEGs. MiRNAs—blue squares; TFs—green circles; DEGs—yellow circles; red line—connection between TF and DEGs; gray line—connection between miRNAs and DEGs.

Our study utilized the miRNet online platform to identify micro RNAs (miRNAs) and transcription factors (TFs) capable of regulating genes pivotal in cholesterol metabolism. This research used a human, rather than a rat, database to conduct the miRNA examination due to limitations within the software, which does not support a transcription factor database for rat samples. As a result, human data were selected as the most viable alternative to accurately investigate the miRNA pathways and interactions under study. We uncovered several miRNAs interacting with identified key genes, including miRNA-1-3p, miRNA-29a-3p, miRNA-29b-3p, miRNA-29c-3p, miRNA-31-3p, miRNA-34a-5p, miRNA-92a-3p, miRNA-124-3p, miRNA-155-5p, and miRNA-192-5p, all of which interacted with *PCSK9*, *SCD1*, and *DHCR24*. Furthermore, we identified TFs exerting regulatory control over these genes. Sterol regulatory element binding transcription factor 2 (SREBF2) was found to regulate both *PCSK9* and *HMGCR*. Additionally, sterol regulatory element binding transcription factor 1 (SREFB1) and hepatocyte nuclear factor 4 alpha (HNF4A) interacted with *PCSK9*, while transcription factor Sp1 regulates *DHCR24* and retinoic acid receptor alpha (*RARA*) interacts with *SCD1* (Figure 9).

## 4. Discussion

Our investigation was conducted as a part of a study by Leontowicz et al. (2016) [17]. In earlier steps, the bioactivity and nutritional properties of kiwifruits were examined [16]. Then, an in vivo experiment was performed, and blood and tissue samples were used to analyze the impact of kiwifruit supplementation on lipid metabolism and other metabolic indices in rats with induced hypercholesterolemia [17]. Blood was collected to determine cholesterol and other blood biomarkers, which showed statistically significant changes in lipid profile and atherogenic indices. Sections of the liver and aorta were examined. Cholesterol affects liver mass and lipid distribution. Some kiwifruit-fed groups had lower rates of hepatic steatosis and fewer aortic lesions, including ChGENE and ChHAYW, which were selected for gene expression analysis. In addition, analyses of hematological parameters, coagulation indices, markers of inflammation, total antioxidant capacity, liver enzymes, as well as amylase, lipase, glucose, and albumin were also performed and the results are described and discussed in Leontowicz et al. (2016) [17].

### 4.1. The Effect of ChGENE/ChHAYW Common Genes in Hypercholesterolemia

In this study, we analyzed gene expression in the livers of rats subjected to an atherogenic diet (1% cholesterol). The rats were supplemented with either 5% freeze-dried *Actinidia arguta* (cultivar Geneva) kiwifruit or 5% freeze-dried *Actinidia deliciosa* (cultivar Hayward) kiwifruit (Figure 1). Our gene expression analysis between the kiwifruit groups, ChGENE and ChHAYW, revealed only two common DEGs: downregulated *PCSK9* and upregulated *CGREF1*. Interestingly, in the case of Geneva kiwifruits, we observed significant expression differences in 72 genes (Figure 2, Figure 3 and Figure 4; Supplementary File S1).

Among the common genes for ChGENE and ChHAYW kiwifruit cultivars, *PCSK9* has been identified. *PCSK9* plays a pivotal role in lipid metabolism and hepatic function, with significant implications for NAFLD. The first fact to note about *PCSK9* is its role in modulating the amount of LDL receptors on the liver cell surface. The study proves that *PCSK9* enhances the degradation of LDL receptor proteins in the liver. Knocking down *PCSK9* in mice reduces plasma cholesterol by increasing hepatic LDL receptors and the rate of LDL cholesterol leaving circulation [22]. Our study revealed that freeze-dried Geneva and Hayward kiwifruit cultivars could downregulate *PCSK9* gene expression and possibly have the ability to reduce cholesterol plasma levels, inhibiting lipid accumulation in the liver. We showed that *PCSK9* is involved in several pathways, including VLDLR internalization and degradation, LDL clearance, plasma lipoprotein assembly, remodeling, and clearance, as well as post-translational protein phosphorylation (Figure 6 and Figure 7). Furthermore, *PCSK9* is linked with various genes, such as *APOE* (indirectly, by promoting degradation of LDLR [23]), *LDLR* (internalization and degradation [24]), *VLDLR* (degradation [24]), and *SIRT6* (suppression of *PCSK9* expression [25]). However, our study did not identify their changed expression (Figure 8). These findings align with the results of Poirier et al., who also demonstrated *PCSK9* efficacy in degrading not only LDLR but also closely related family members VLDLR and APOER2. These receptors are pivotal in lipid metabolism and neuronal development, especially familial hypercholesterolemia. This underscores the multifaceted nature of *PCSK9* involvement in critical biological processes related to liver steatosis [26]. In medium-chain fatty acid (MCFA)-fed pigs, increased *PCSK9* activity likely decreases cholesterol uptake into muscle cells, resulting in higher circulating cholesterol levels [27].

The second DEG common to the supplementation of Geneva and Hayward kiwifruit varieties was *CGREF1*, which was upregulated in both treatments. While *CGREF1* is not explicitly identified as a critical player in lipid metabolism, its protective roles in cell proliferation and growth suggest it could help mitigate some harmful effects of lipid accumulation in the liver [28]. Moreover, some reports indicate that *CGREF1* can prevent excessive apoptosis [28], which could be critical in liver tissue repair and regeneration processes. By promoting cell growth and inhibiting unnecessary cell death, *CGREF1* helps maintain liver tissue integrity after cholesterol-induced damage and encourages regeneration, preventing liver fibrosis or damage progression. Some miRNAs were predicted as possibly impacting *CGREF1* activity (Figure 9). MiR-212-3p and miR-155-5p may suppress the protective effects of *CGREF1*, leading to the deterioration of liver diseases like NAFLD, where maintaining hepatocyte integrity is crucial for preventing disease progression. On the contrary, miR-29c-3p may support *CGREF1* activity, contributing to improved liver regeneration and reduced fibrosis or inflammation, thus potentially protecting against lipid accumulation and liver damage. The balance of these miRNAs in liver cells could critically determine whether *CGREF1* can exert its protective effects on the liver. Although *CGREF1* is not a factor considered crucial for processes involving lipid and cholesterol metabolism, its identification as one of two genes with altered expression common to the Geneva and Hayward kiwifruit varieties suggests that the activity of this gene may be key to reducing hepatic steatosis in response to the use of freeze-dried kiwifruit containing antioxidants, fiber, and vitamins as supplements in a high-cholesterol diet.

### 4.2. The Effect of Geneva Kiwifruit Supplementation

Leontowicz et al. (2016) indicated that reducing hepatic steatosis was most apparent when a 1% cholesterol diet was supplemented with the Geneva kiwifruit, even when compared to the supplementation of the Hayward kiwifruit cultivar [17]. Thus, the significant reduction of lipid accumulation in the liver in animals in the ChGENE group does not depend on the activity of the previously mentioned genes *PCSK9* and *CGREF1*. In addition, 70 DEGs were identified for the ChGENE-Ch comparison, several of which were identified as hub genes (Figure 8).

#### 4.2.1. Reduced Biosynthesis of Cholesterol

A study by Leontowicz et al. (2016) showed statistically lower blood serum LDL concentrations in ChGENE and ChHAYW rats compared to the Ch group and significantly lower hepatic steatosis for both groups, with liver tissue from the ChGENE group closely resembling that of the negative control group (normal liver without cholesterol loading) [17]. Two of the identified genes with downregulated expression in the liver of ChGENE relative to a Ch control diet containing 1% cholesterol were *HMGCR* and *DHCR24*. *HMGCR* is the rate-limiting enzyme in cholesterol synthesis and is pivotal in maintaining cholesterol balance. An increase in *HMGCR* expression leads to heightened cholesterol synthesis, whereas its inhibition decreases cholesterol production in the liver. Inhibition of the activity of this gene, and thus of cholesterol synthesis, has found application in the treatment of hypercholesterolemia. Lovastatin, a hypolipidemic drug, can curl downstream cholesterol synthesis by inhibiting *HMGCR* [29]. In a study involving pigs, a specific *HMGCR* polymorphism (HMGCR:c.807A.C) was identified as having an association with serum LDL-bound cholesterol levels and exhibiting a negative correlation with serum HDL levels [30]. In rats with hyperlipidemia induced by a high-fat diet, there is a significant increase in the expression of the HMGCR protein; however, following treatment with atorvastatin ester, the expression of the HMGCR protein decreases significantly [31]. Our study confirms that the *HMGCR* gene, downregulated in the ChGENE group, may change its expression when exposed to kiwifruit supplementation and thus possibly help reduce cholesterol levels, contributing to lower liver fat accumulation.

The second gene that may partner with *HMGCR* in reducing liver exposure to steatosis resulting from a high cholesterol supply is *DHCR24*. It plays a pivotal role in regulating cholesterol homeostasis by controlling cholesterol synthesis. The deficiency of *DHCR24* activity leads to reduced cholesterol levels in both the plasma membrane and intracellular compartments. Knockdown of *DHCR24* results in the inhibition of cholesterol synthesis and a decrease in the plasma membrane cholesterol content [32]. Zhou et al. (2023) reported a novel strategy to activate liver X receptors (*LXR*) via pharmacological *DHCR24* inhibition by SH42 to prevent diet-induced hepatic steatosis and liver inflammation for the treatment of NAFLD [2]. However, Jin, Yang et al. (2024) indicate reduced expression of both genes mentioned above when a high-cholesterol diet is consumed by zebrafish accompanied by hepatic steatosis. The authors point to a reduction in de novo cholesterol synthesis in the face of reduced expression of these genes caused by cholesterol accumulation in the blood [33]. In our study, the expression of *HMGCR* and *DHCR24* was significantly lower when a freeze-dried Geneva cultivar was used, with concomitant low serum cholesterol levels [16], unlike in zebrafish, where plasma cholesterol levels were high. Our findings suggest that kiwifruit supplementation may induce a mechanism to lower the expression of these genes and cholesterol biosynthesis despite low serum cholesterol levels. This potential effect of kiwifruit on gene expression could have significant implications for treating metabolic diseases, providing a promising avenue for future research and possible therapeutic interventions.

#### 4.2.2. Lipid Transport

Solute carrier family 27 member 5 (*SLC27A5*) plays a pivotal role in lipid metabolism, acting as a key fatty acid transporter. Its primary function is facilitating the uptake of long-chain fatty acids into cells, particularly in the liver. This process is crucial for maintaining lipid homeostasis and providing substrates for lipid metabolism [34]. *SLC27A5* serves as a critical regulator of lipid metabolism, orchestrating this process through interactions with various enzymes and transcription factors. The declined activation of this gene in our study aligns with prior research. For instance, a decrease in the expression of *SLC27A5* has been linked to the inhibition of lipid synthesis [35]. Furthermore, knockdown of *SLC27A5* in HEPG2 cells resulted in decreased lipid synthesis and tyrosine metabolism, leading to activation of the cell cycle and increased cell proliferation [36]. *SLC27A5*, also known as *FATP5*, is associated with progression and hepatic fat loss in NAFLD patients, in whom a reduction in hepatic *FATP5* expression is inversely correlated with histological markers of progression. Furthermore, *FATP5* regulates fatty acid intake and transport within the liver. In advanced NASH patients, diminished hepatic *FATP5* expression might contribute to the observed hepatic fat loss, positioning *FATP5* as one of the genes involved in regulating proteins related to fatty acid metabolism [37]. In addition, the targeted deletion of *FATP5* in mice led to a notable decrease in serum cholesterol levels, suggesting that this change may indirectly affect cholesterol metabolism [34]. Thus, the reduced serum cholesterol levels described by Leontowicz et al. (2016) [17] may be the result, at least in part, of identified in our study the reduced activity of this gene in the ChGENE group.

Among DEGs, two upregulated genes are of particular interest in light of decreased steatosis of rats consuming an atherogenic diet supplemented with freeze-dried Geneva cultivar [17], namely ATP-binding cassette, sub-family G, member 3 (*ABCG3*) and ATP-binding cassette, subfamily G (WHITE), member 3-like 4 (*ABCG3L4*). The *ABCG3* gene is a member of the ATP-binding cassette (ABC) transporter family, which is known for using hydrolysis to provide the energy needed to translocate substrates across cellular membranes [38]. Most cell types in the body have a very limited ability to catabolize cholesterol, making cholesterol efflux essential for cholesterol homeostasis. One of the mechanisms involved in cholesterol efflux is ABC transporter activity, particularly *ABCA1* and *ABCG1*. *ABCA1* is critical for cholesterol and phospholipid efflux into apolipoprotein A-I and HDL biogenesis. It was proven by Moradi et al. (2021), who observed that upregulation of this gene in hamsters fed with a high-fat diet supplemented with kiwifruit improves cholesterol efflux from the liver [39]. *ABCG1* promotes cholesterol efflux mainly to HDL particles [40]. *ABCG3* is only found in rodents, and its function is poorly understood [41]. According to our results and its upregulation in liver tissue of the ChGENE group, it likely facilitates the transport of lipids, such as cholesterol, out of cells. This process helps maintain lipid balance and prevents intracellular lipid accumulation, as reflected by the lack of hepatic steatosis in rats representing this group [17]. Although less studied, *ABCG3L4* seems to function similarly to *ABCG3*, given that it belongs to the same family of ABC transporters and due to its functional annotations. It likely plays a role in lipid efflux, particularly cholesterol and other fatty molecules, to maintain a healthy liver. By removing excess lipids, *ABCG3* and *ABCG3L4* help prevent lipid overload in cells, reducing the risk of lipid-related pathologies such as fatty liver or atherosclerosis. Upregulation of these genes likely enhances lipid transport and efflux activities, promoting the reduction of intracellular lipid levels and contributing to cellular lipid homeostasis.

#### 4.2.3. Anti-Inflammatory and Regenerative Effects

The role of other DEGs, such as programmed death-ligand 1 gene (*CD274*; *PD-L1*), ephrin type-A receptor 4 (*EPHA4)*, and Protocadherin 7 (*PCDH7*), in modulating inflammatory and regenerative processes is crucial for understanding the molecular mechanisms that support liver health, particularly in atherogenic diets that induce liver damage and systemic inflammation [42]. Recent studies by Leontowicz et al. (2016) highlight the potential of dietary interventions, such as the supplementation of freeze-dried kiwifruit, to mitigate these harmful effects [17]. *CD274* plays a pivotal role in regulating immune responses. Its primary function is to inhibit T-cell activity by binding to the PD-1 receptor and suppressing inflammatory pathways. In an atherogenic diet, upregulation of *CD274* in rats supplemented with freeze-dried Geneva kiwifruit may suggest an enhanced ability to modulate immune responses. By reducing excessive immune activation, *CD274* may help limit liver inflammation, preventing immune-mediated tissue damage. This anti-inflammatory effect is crucial in preserving liver function and mitigating the progression of diet-induced liver steatosis. The freeze-dried kiwifruit’s polyphenolic content, known for its antioxidant and anti-inflammatory properties, may drive this upregulation [16,17].

Upregulated in the ChGENE group, *EPHA4*, a member of the Eph receptor family, is involved in numerous biological processes, including tissue remodeling, neuronal development, and cell migration [43,44]. Its upregulation in rats fed an atherogenic diet supplemented with freeze-dried Geneva kiwifruit suggests that this gene may promote liver tissue repair and regeneration. Cholesterol-rich diets are known to induce liver damage through oxidative stress and lipid accumulation, resulting in the need for enhanced regenerative mechanisms [45,46]. The ability of *EPHA4* to respond to dietary modulation through kiwifruit supplementation could be linked to its bioactive compounds, such as vitamin C and polyphenols, which may stimulate regenerative pathways and improve overall liver health [16].

One of the upregulated ChGENE group DEGs is *PCDH7*, a member of the protocadherin family, proteins primarily involved in cell adhesion [47]. While its function is characterized mainly in neural tissues [48], *PCDH7* upregulation in rats fed an atherogenic diet supplemented with Geneva kiwifruit indicates its potential role in liver tissue integrity and regeneration. Strengthening cell adhesion may help maintain the structural integrity of hepatocytes, supporting tissue recovery and reducing the likelihood of fibrosis [49]. Additionally, its involvement in immune modulation [50,51] could further enhance liver repair by promoting an environment conducive to healing while limiting excessive inflammation. Kiwifruit’s rich nutrient profile likely contributes to this upregulation, supporting the hypothesis that dietary antioxidants and bioactive compounds can positively influence genes involved in tissue regeneration and repair.

The upregulation of the genes mentioned above in the ChGENE group underscores the potential of dietary interventions in modulating genes associated with inflammation and tissue regeneration. Their role in suppressing immune-mediated liver damage, tissue remodeling, liver regeneration, cell adhesion, and tissue integrity highlights their importance in preserving liver health under stress conditions. This suggests that with its bioactive components, kiwifruit may offer a protective effect against diet-induced liver damage. Further studies are needed to elucidate these genes’ precise molecular mechanisms and therapeutic potential in liver diseases.

#### 4.2.4. Altered Glucose and Fatty Acid Metabolism

Among the identified hub genes, one of the most noteworthy is downregulated *SCD1*. It is a pivotal enzyme in fat biosynthesis, particularly in converting saturated fatty acids (SFAs) into monounsaturated fatty acids (MUFAs). *SCD1* is critical in governing the balance between unsaturated and saturated fatty acids, a balance of significance in cell signaling, maintaining membrane fluidity, and facilitating various essential physiological processes [52]. It catalyzes the transformation of oleate and palmitoleate, the fatty acids integral to different lipids, encompassing phospholipids, triglycerides, and cholesteryl esters [53]. Research findings indicate that a deficiency in *SCD1* results in decreased body adiposity and heightened insulin sensitivity. Conversely, an overexpression of *SCD1* is associated with metabolic conditions such as diabetes and non-alcoholic fatty liver disease [54]. This implies that *SCD1* actively participates in lipid metabolism and contributes to the emergence of hyperlipidemia [55]. This study shows that the Geneva kiwifruit cultivar can lower *SCD1* expression, decreasing liver lipid accumulation. These findings are consistent with the results presented in the Major CA (2008) study, which found that when *SCD1* activity was inhibited in cholesterol-fed hamsters, it led to decreased body weight, reduced adipose tissue mass, and improved feed efficiency. In addition, inhibiting *SCD1* activity affected the expression of genes related to liver lipid metabolism. In mice, the knockout of the *SCD1* gene resulted in the downregulation of hepatic lipogenic genes and a reduction in hepatic SREBP1C expression [56]. Pathway analysis indicates that *SCD1* is linked to the enrichment of specific pathways, including the activation of gene expression by SREBF, regulation of cholesterol biosynthesis by SREBF, metabolism of steroids, and the regulation of gene expression associated with lipogenesis by NR1H2 and NR1H3. These pathways play a crucial role in cholesterol metabolism. Furthermore, a literature analysis revealed a connection between *SCD1* and the fatty acid synthase gene (*FASN*). Both enrich the activation of gene expression by SREBF pathways. It is worth noting that SREBP1C appears to target genes primarily involved in fatty acid synthesis, while SREBP2 seems to focus on genes related to cholesterol synthesis [57]. These interactions suggest/confirm the engagement of identified in our study genes in cholesterol metabolism.

Another identified DEG, the *PRODH1* gene, is essential in proline catabolism, which affects various metabolic processes such as lipid metabolism. *PRODH1* is involved in proline oxidation to pyrroline-5-carboxylate in mitochondria and may influence lipid metabolism by affecting oxidative stress and energy balance [58,59]. *PRODH1* deficiency or mutations have been shown to cause defects in mitochondrial metabolism, particularly β-oxidation of fatty acids and accumulation of lipid intermediates [60]. These abnormalities may be associated with metabolic disorders, including obesity and hepatic steatosis, disruption of lipid homeostasis, and cancer [61,62]. *PRODH1* was also shown to be AMPK pathway-dependent, a cellular energy and glucose status monitor. The direct regulatory effect of PPARG, which regulates fatty acid storage and glucose metabolism, on *PRODH1* was also confirmed [61]. Its downregulation in our study may affect amino acid metabolism, which is linked to broader glucose and lipid metabolism pathways.

Further supporting the altered lipid metabolism observed in metabolic disorders, the solute carrier family 2 member 1 gene (*SLC2A1*; *GLUT1*), responsible for glucose transport [63], was downregulated under the influence of the Geneva kiwifruit cultivar. Downregulation decreases glucose availability for lipid synthesis and hence may limit fat accumulation in the liver of the ChGENE group. This downregulation of *SLC2A1* may be seen as a protective adaptation against an excess cholesterol supply because a high cholesterol supply is known to result in conditions like NAFLD. Also, in this respect, results showed that *FGF9*, responsible for cellular growth and lipid metabolism processes [60], was downregulated in our study. This growth factor is involved in regulating fatty acid synthesis and energy metabolism. Its reduction might give rise to altered synthesis and metabolism of lipids, further contributing to dysregulation in metabolic pathways responsible for glucose and fatty acid metabolism [64]. These interrelated mechanisms further indicate that changes in glucose and lipid metabolism due to the described genes above can be central in liver steatosis, suggesting that freeze-dried Geneva kiwifruit could be a promising dietary intervention to reduce lipid accumulation in the liver.

#### 4.2.5. Role of Olfactory Receptors

Special attention must be paid to a group of downregulated DEGs: members of olfactory (ORs) and vomeronasal receptors (VNRs) (Supplementary File S1). ORs and VNRs transduce chemical signals into electrical signals by interacting with G-proteins and triggering downstream signaling pathways [65]. They are primarily known for detecting odorants and pheromones [66]. However, recent research has begun to uncover their potential involvement in metabolic processes, including lipid metabolism in the liver [67,68]. They are expressed in various tissues beyond the olfactory epithelium, including the liver and adipose tissue. ORs can be activated by specific lipids and fatty acids, which may trigger signaling pathways related to lipid metabolism. For example, activating specific ORs can influence the expression of genes such as acetyl-CoA carboxylase (*ACC*), *FAS*, and *SCD1* involved in lipogenesis and lipolysis [68]. One of the vomeronasal receptors, *VNR66*, is part of a family of receptors responsive to pheromones; it may also affect metabolic regulation. *VNR66* might be involved in sensing changes in lipid levels or other metabolites, which could lead to adaptations in metabolic pathways in the liver. This could include modulating the balance between lipid synthesis and degradation. Metabolic sensors like AMPK and mTOR, which react to variations in ATP, glucose, and other metabolites like lactate or acetyl-CoA, can detect changes in cellular surroundings [69,70].

The exact mechanisms by which olfactory and vomeronasal receptors influence lipid metabolism in the liver are still being elucidated. ORs and VNRs may initiate signaling cascades that influence critical metabolic pathways. This includes pathways governed by hormones such as insulin and glucagon, which regulate lipid metabolism [68,71]. In the case of our study, a reduction in the expression of these genes seems to be beneficial from the point of view of reducing hepatic steatosis. Such an effect was exerted on the activity of ORs and VNRs by adding a lyophilized Geneva kiwifruit cultivar. The interplay between sensory perception and metabolism presents a fascinating study area with implications for understanding energy balance and metabolic diseases.

### 4.3. The Prediction of miRNA and TF Interaction with Hub Genes

The prediction of interactions between miRNAs and TFs with hub genes is essential for understanding the complex regulatory networks that govern cellular processes such as lipid metabolism. This approach is important in identifying how regulatory networks are coordinated to maintain cellular homeostasis and how their dysregulation contributes to pathological conditions [72]. The following interactions were identified in our study (Figure 9).

MiRNA-29 is pivotal in regulating lipid metabolism, particularly in hepatic cells. Overexpression of miRNA29d-3p significantly inhibited genes crucial for triglyceride synthesis, such as diacylglycerol O-acyltransferase 1 (*DGAT1*) and glycerol-3-phosphate acyltransferase, mitochondrial (*GPAM*), in bovine mammary epithelial cells (BMECs). This upregulation led to a notable decrease in triglyceride levels, highlighting the miRNA involvement in lipid accumulation regulation. Moreover, Zhao et al. (2021) showed that overexpression of miRNA29d-3p decreased the expression of genes associated with fatty acid synthesis and desaturation, such as *FASN*, *SCD1*, *ACC*, and the transcription factor SREBF1. Conversely, interference with miRNA-29d-3p boosted the expression of lipid metabolism-related genes and raised triglyceride concentration in the cells. A luciferase reporter assay confirmed *GPAM* as a direct target of miRNA-29d-3p, shedding further light on its role in lipid metabolism regulation [73]. In addition, heightened levels of miRNA-29 species in the liver are evident in insulin resistance and type 2 diabetes models. Inhibiting miRNA-29 has demonstrated improvements in hepatic insulin resistance, highlighting its role in liver lipid metabolism modulation [74]. Overexpression of miRNA-34a diminishes apoptosis in liver cells, potentially influencing lipid metabolism. The decreased apoptosis rates observed in cells with elevated miRNA-34a levels could play a role in enhancing lipid homeostasis [75].

MiRNA-31-5p plays a significant role in regulating genes associated with lipid metabolism, and this miRNA is identified as an essential regulator of lipid metabolism-related genes in the intestine, such as 3-hydroxy-3-methylglutaryl-CoA synthase 2 (*HMGCS2*), acetyl-CoA acetyltransferase 1 (*ACAT1*), and oxidized low-density lipoprotein receptor 1 (*OLR1*). Moreover, miRNA-31-5p and other miRNAs, such as miRNA-99b-5p and miRNA-200a-5p, have been identified as critical regulators of lipid metabolism-related genes in the intestines [76].

SREBF2 functions as a transcription factor overseeing genes related to cholesterol metabolism. It orchestrates the activity of critical genes like *PCSK9* and *HMGCR*, which are pivotal in lipid processing. *PCSK9* attaches to LDL receptors, accelerating their breakdown and elevating LDL cholesterol levels in circulation. SREBF2 can boost *PCSK9* expression, exacerbating LDL cholesterol levels and perturbing lipid metabolism. *HMGCR* is the key enzyme governing cholesterol synthesis and is the target of statins for cholesterol control. SREBF2 governs *HMGCR* expression, influencing cholesterol production and lipid metabolism [77].

## 5. Conclusions

Our study reveals that kiwifruit lyophilizes, particularly the *Actinidia arguta* Geneva cultivar, can effectively modulate gene expression related to cholesterol metabolism in rats fed an atherogenic diet. The upregulation of *CGREF1* and its association with the downregulation of *PCSK9* suggests a role in reducing liver cholesterol. Additionally, the downregulation of *SCD1* indicates a potential mechanism for liver fat reduction. Kiwifruit consumption also influenced genes like *HMGCR*, *DHCR24*, and *SLC27A5*, which are crucial in cholesterol metabolism and showed connections with liver-expressed miRNAs. These findings suggest that dietary supplementation with kiwifruit may be a promising strategy to manage cholesterol-related conditions and protect the liver from steatosis. However, it is important to note that this study was conducted on rats, and further research is needed to determine the effects of kiwifruit on human health.

## Figures and Tables

**Table 1:** List of primers used for real-time qPCR.

Gene Symbol	Accession Number	Forward Primer	Reverse Primer	Annealing Temp. (°C)	Annealing Time (s)	PCR Product Length
*DHCR24*	NM_001080148	5′AAGGGGTTGGAGTTCGTTC3′	5′TGAGACAGTGAGCCATCCA3′	58	15	243
*HMGCR*	NM_013134	5′GGACTGAAACACGGGCATT3′	5′AACACGGCACGGAAAGAAC3′	58	15	197
*PCSK9*	NM_199253	5′GTGTGTGTGGCACGAATCC3′	5′AAGTTCCCCCAGGCAGAGT3′	58	15	224
*PRODH*	NM_001135778	5′CGCAGGTTCAATGTGGAT3′	5′GGCATTGGTGGCTTCATA3′	58	15	222
*SCD1*	NM_139192	5′CACACTGGTGCCCTGGTA3′	5′GGGAAGGCGTGATGGTAG3′	58	15	225
*GAPDH*	NM_017008	5′CCCACACTGTGCCCATCTAT3′	5′AAGGGTGTAAAACGCAGCTC3′	60	15	198

## Data Availability

The data obtained from the microarray experiment were deposited in the National Center for Biotechnology Information Gene Expression Omnibus database (NCBI GEO) with accession number GSE79715.

## Supplementary Materials

The following supporting information can be downloaded at: https://www.mdpi.com/article/10.3390/nu16233999/s1, Supplementary File S1: List of differentially expressed genes (DEGs).
